# An electron microscopic study of the archaeal feast/famine regulatory protein: 3. Labeling of the N-terminus using nickel

**Published:** 2004-02-01

**Authors:** Sanae A. Ishijima, Lester Clowney, Hideaki Koike, Masashi Suzuki

**Affiliations:** *) National Institute of Advanced Industrial Science and Technology (AIST), AIST Tsukuba Center 6-10, 1-1-1, Higashi, Tsukuba, Ibaraki 305-8566, Japan; **) Japan Science and Technology Agency (JST), Core Research for Evolutionary Science and Technology (CREST) Honmachi, 4-1-18, Kawaguchi Center Building, Kawaguchi, Saitama 332-0012, Japan

**Keywords:** Color electron microscopy, cryo-electron microscopy, EELS, electron spectroscopic imaging, energy filtration

## Abstract

Histidine residues added to the N-terminus of a polypeptide (i.e. a His-tag) was used, for the first time to our knowledge, for electron labeling of the protein upon its electron spectroscopic imaging. Originally such a His-tag was developed by another group to purify modified proteins by taking advantage of their affinity to nickel. The feast/famine regulatory protein pot0434017 (FL11) was modified by adding six His residues to its N-terminus, so that each His pair would chelate a nickel ion. An electron microscope was operated at 200 KeV, and the electrons that lost the energy by ~875 eV upon interaction with the metal were selectively focused. The majority, 60–70%, of the spots detected in the electron micrographs were paired by distances shorter than 80 Å, and over 70% of them were paired by distances shorter than 40 Å. It is concluded that the protein molecules formed dimers, and the termini of most of the protein molecules were labeled with nickel by this method.

## Introduction

**Fig. 1:**
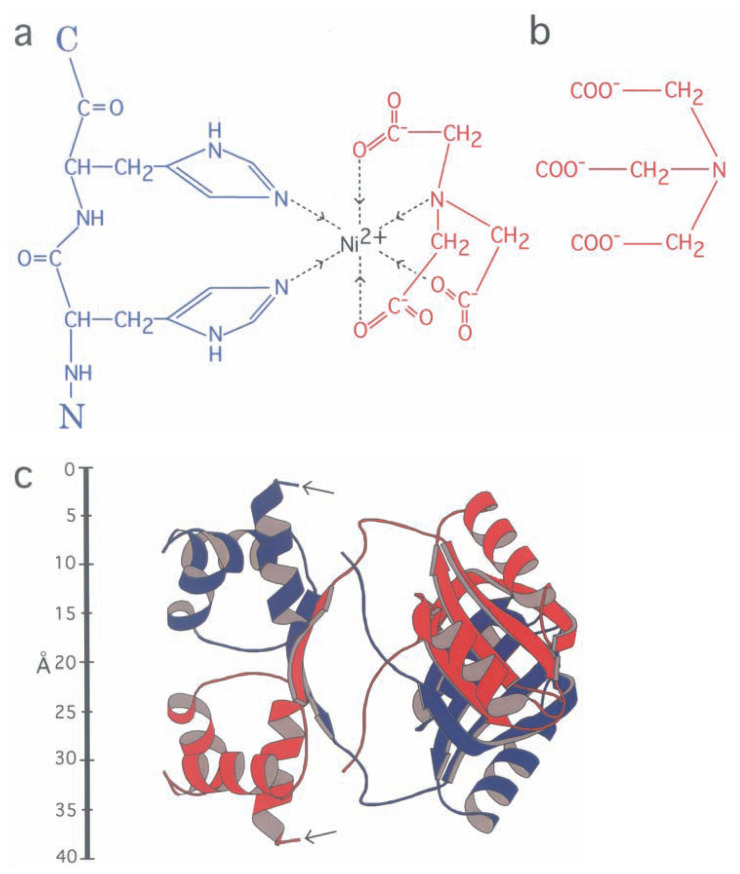
Labeling of the N-terminus of FL11 using interaction between His residues and nickel. (a) A schematic drawing of chelation of a nickel ion (black) by two histidine residues in the polypeptide (blue, the N-C direction indicated) and a single molecule of nitrilotriacetic acid (NTA, red). (b) The chemical structure of NTA at a basic pH. (c) A ribbon diagram of a dimer of FL11, determined by X-ray crystallography.6) In this structure the coordinates of four residues at the N-terminal end are missing, and thus each N-terminus in this diagram starts with the fifth residue (indicated by an arrow).

When proteins are artificially expressed using *E. coli*, extra histidine residues are often introduced to their N- or C-termini, and these “His-tags” are used for purification of the proteins.1) A pair of His residues can chelate a nickel ion (Fig. 1a left), by occupying two of the six molecular orbits around the metal available for coordinate bonding (e.g. ref. 2)). In an aqueous solution, the remaining four orbits are occupied by water molecules. These water molecules can be replaced (Fig. 1a, right) by a stronger coordinator such as nitrilotriacetic acid (NTA, Fig. 1b). Thus, NTA conjugated to a matrix such as agarose will selectively bind these tagged proteins, which can be eluted by adding a high concentration of imidazole.1)

In this study, a His-tag was used for a purpose different from the original one, i.e. electron labeling of a protein examined under an electron microscope. Using an energy filtration device, it is possible to selectively focus electrons, which have interacted with a particular element such as nickel,3) i.e. electron spectroscopic imaging, electron energy-loss spectroscopy (EELS) or color electron microscopy.4),5) The protein labeled in this study was the feast/famine regulatory protein (FFRP) pot0434017 (FL11) from the hyper-thermophilic archaeon *Pyrococcus* sp. OT3. The classification FFRPs encompasses archaeal DNA-binding proteins with the *E. coli* transcription factors Lrp (the leucine-responsive regulatory protein) and AsnC (the asparagine synthase *C* gene product).5)–13) These proteins, in general, have multiple assembly forms. The structural unit of FL11 is a dimer, and by assembling such dimers, higher-order structures, disks or helical cylinders are formed.6),10),14)

## Loss of electron energy upon interaction with nickel

**Fig. 2:**
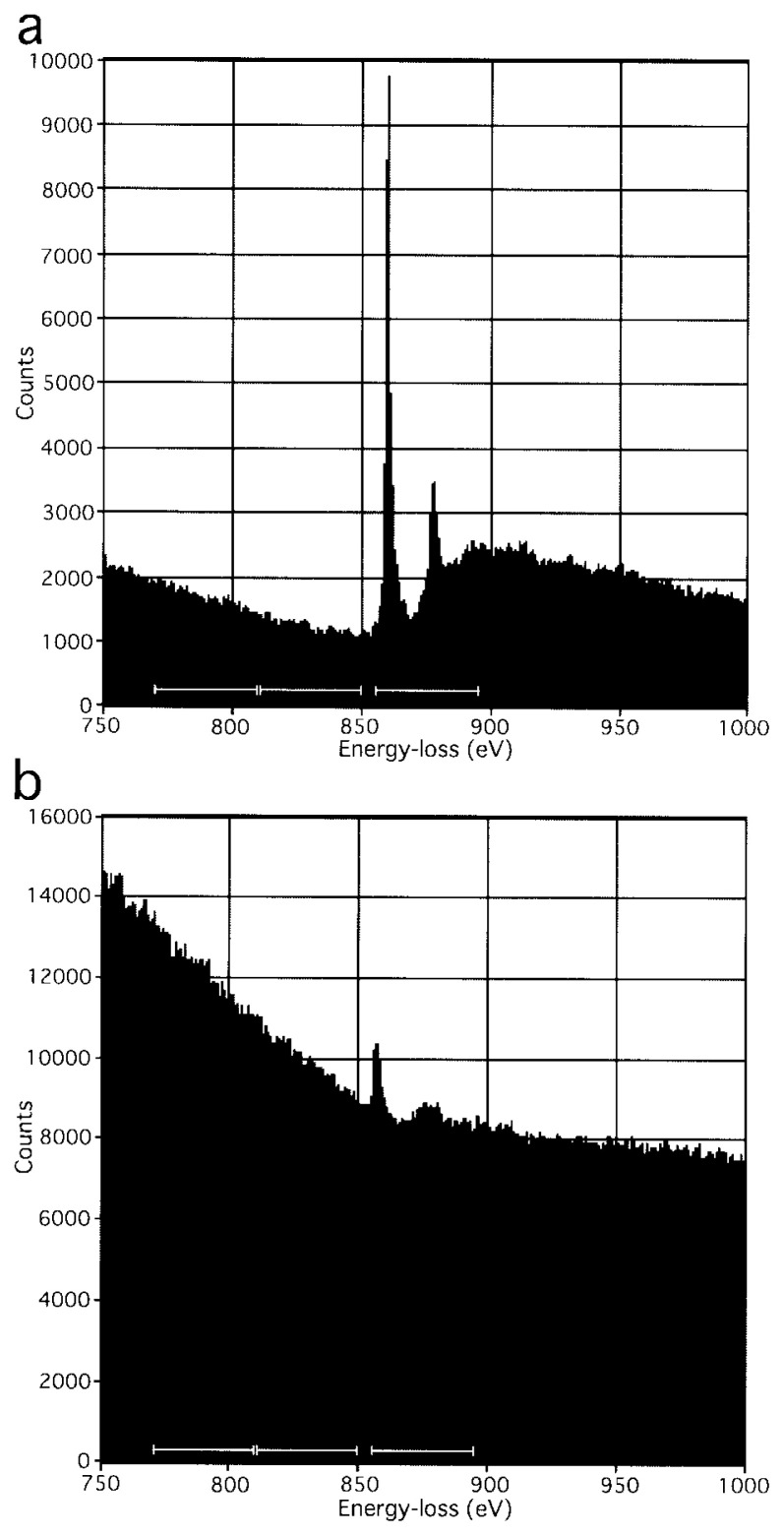
Electron energy-loss spectra measured using a film of nickel oxide, in the absence (a) or presence (b) of amorphous ice. On the basis of these spectra, electrons, that had lost an energy of 855–895 eV, were selectively focused to images (Fig. 3), where the background was calculated by the “two area” method (see 4.4.2 of ref. 3)) using electrons that had lost energies by 770–810 eV and 810–850 eV, respectively, and subtracted (the three energy ranges indicated by bars). Note the large plasmon loss in (b) present at lower loss-energies, due to amorphous ice.

A thin film of NiO15) was made. Collodion-coated copper grids (150 mesh) were strengthened by evaporating carbon. After glow-discharging these grids for aquiring better hydrophilicity, a droplet, 10 μl, of a NiO solution (500 mg/ml) was laid on each grid. After a few minutes, the droplet was removed using a piece of filtration paper. The grid, still slightly wet, was left overnight and dried by air. An electron microscope (Tecnai F20, FEI) was operated at 200 KeV, and the electron energy-loss spectrum of the film (Fig. 2a) was recorded using an energy spectrometer (GIF200, Gatan). The highest peak was found at around a loss of 875eV, which is consistent with the literature.15) A similar spectrum (Fig. 2b) was measured in the presence of amorphous ice (see another section, Electron spectroscopic imaging).

Upon interaction of mono-energetic electrons with a specimen, the energy of some of the electrons remains the same, i.e. elastic or zero-loss scattering, which involves Coulomb interactions with the nucleus of the atom. The other electrons loose the energy by varying values, i.e. inelastic scattering. In one type of energy losing, the lost energy, e.g. ~875 eV, is absorbed by an electron inside the atom. This process is characteristic of the element, in this case, nickel; the core-loss.3) By selecting electrons of different energies, a multiple number of images can be obtained, showing distributions of different elements in the same specimen, i.e. electron spectroscopic imaging or color electron microscopy.4), 5)

## Labeling of FL11 using nickel

The protein FL11 was expressed in a modified form and purified by the method previously described6),10): upon expression of the protein six additional histidine residues in the sequence MGSSHHHHHHSSGLVPRGSH were added to its N-terminus (i.e. a His-tag) using the pET28a vector (Novagen). The modified protein, 2 μM in 10 mM Tris-HCl buffer (pH = 8.0) containing 130 mM KCl, was mixed with 10 mM NiSO_4_ in the absence or presence of 8 mM nitrilotriacetic acid (NTA, Fig. 1b), which was not conjugated to any matrix. After incubation for 30 min at 4 °C, free metal was removed by changing the buffer four times by centrifugation-driven gel filtration, using a gel, Microcon-30 (Amicon).

A pair of His residues can chelate a nickel ion: the maximum number of nickel ions attached to the N-terminus of each FL11 monomer is three. Because of the limited resolution of EM images, it is unlikely that two or three metal ions attaching to the same termini could be differentiated.

In what follows, the two types of Ni-compounds of FL11, made in the absence or presence of NTA, are referred to as FL11-Ni (i.e. FL11-Ni_1–3_) and FL11-Ni-NTA (i.e. FL11-[Ni-NTA]_1–3_), respectively.

## Electron spectroscopic imaging

As before,13),14) in order to protect the protein, the method of cryo-electron microscopy was applied. A droplet, 4 μl, of a protein solution was placed on a piece of Parafilm. A holey carbon-coated 300 mesh copper grid (Electron Microscopy Sciences Co.) was floated on the surface of the droplet. After floating for 90 sec, the grid was quickly frozen in liquid ethane into an amorphous ice state, using a freezing apparatus (EM CPC, Leica). This method is a modification of the originals described by Cyrklaff *et al.*16) and Adrian *et al.*17) The grid was maintained at a near liquid nitrogen temperature using a holder (CT3500, Oxford), while an electron microscope (Tecnai F20, FEI) was operated at 200 KeV.

**Fig. 3:**
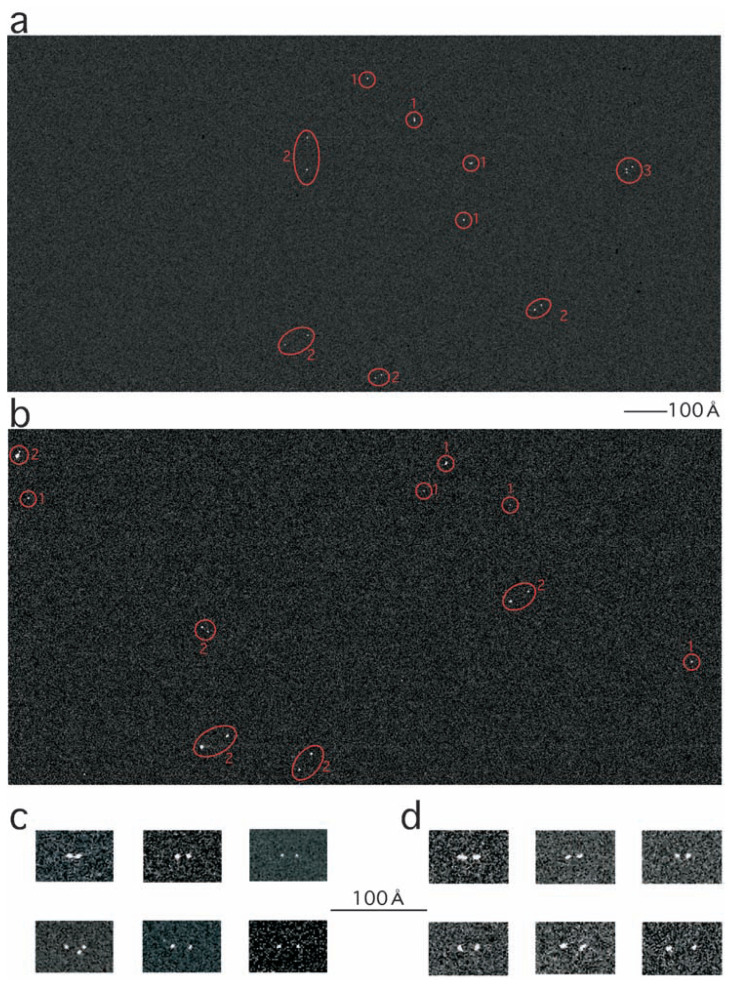
Electron spectroscopic images obtained by selectively focusing electrons that have interacted with nickel. (a) and (b) Half the area covered by the CCD camera are shown; (a) FL11-Ni and (b) FL11-Ni-NTA. Spots identified as paired (2) and singlets (1) are indicated; (3) indicates a cluster of three spots, which contributed to the statistics (Fig. 4) as three pairs. The original magnification of the electron micrographs was × 155 K. The two micrographs are shown at the same scale. (c) and (d) Collections of the “double-eye” images shown at another same scale: (c) FL11-Ni and (d) FL11-Ni-NTA. In one of the images in (c) three spots instead of two are found (bottom left).

Electrons, that had lost an energy of 855–895eV, were selectively focused using an energy filter (GIF200, Gatan), and recorded using a CCD camera (794IF, Gatan). Two more images were recorded by focusing electrons having lost energies by 770–810 eV and 810–850 eV, respectively. Using the “two area” method (see 4.4.2 of ref. 3)), the background was calculated from the last two images in these triplets, and subtracted from the first ones, using the Digital Micrograph software (Gatan). Examples of the electron micrographs obtained are shown in Fig. 3.

## Nickel ions in pairs

Thirteen electron micrographs of FL11-Ni were recorded with a magnification of 155 K. Nineteen micrographs of FL11-Ni-NTA were recorded with the same magnification, but other five micrographs were recorded with another magnification of 63 K. With the magnification of 155 K, each CCD pixel covers an area of 1.54 Å × 1.54 Å. With the magnification of 63 K, it covers an area of 3.79 Å × 3.79 Å. The point resolution of the EM images cannot be better than these pixel sizes. Some other factors, e.g. drifting of the specimen, further restrict the resolution: spots occupying 2–13 pixels each with high intensities were identified as signals.

In the electron micrographs, spots indicating the positions of the nickel ions did not distribute randomly, but they tended to form pairs (Fig. 3). In individual electron micrographs obtained using FL11-Ni, on average 20.8 ± 9.2 spots were found, of which 14.4 ± 7.5 were paired with each other by distances shorter than 80 Å; 69% (Fig. 3a, c). While, in electron micrographs obtained at the magnification of 155 K using FL11-Ni-NTA, 21.2 ± 7.9 spots were found, of which 13.0 ± 5.7 were paired with each other by distances shorter than 80 Å; 61% (Fig. 3b, d). Of these paired spots, either of FL11-Ni or FL11-Ni-NTA, 72–73% were paired with distances shorter than 40 Å. It is likely that these paired spots are images of nickel ions incorporated into dimers of FL11.

**Fig. 4:**
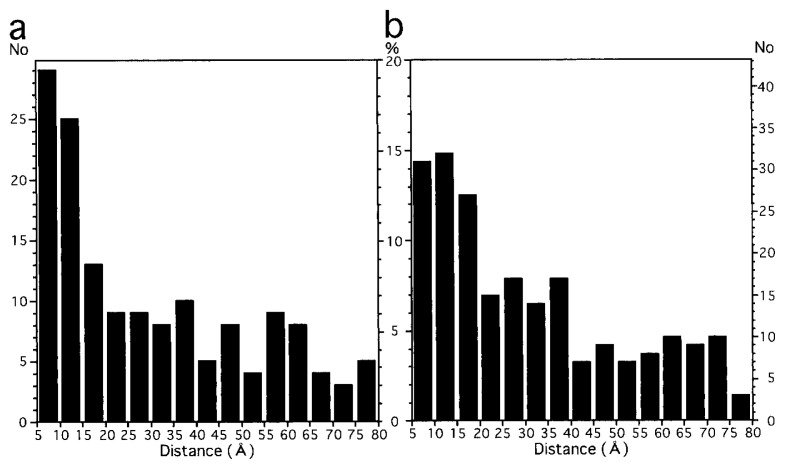
The number of pairs of spots distanced in particular ranges, e.g. 5 ≤ *d* < 10; (a) FL11-Ni and (b) FL11-Ni-NTA. Two types of scales are shown, by the count (outside) and by the percentage (inside).

The number of pairs was found decreasing with increasing the distance between the spots in pairs (Fig. 4). Such a distribution is clearly non-random: for a random distribution, this number should increase instead. The gap between a larger circle of the radius d1 and a smaller circle of the radius d2 has a size, ***π***(d1^2^ − d2^2^) = ***π***(d1 − d2)(d1 + d2). For a random distribution, this size is proportional to the number of spots distanced by d1 to d2 from another spot positioned at the center. While keeping (d1 − d2) constant and increasing (d1 + d2)/2, the size of the gap and thus the number of spots found there should linearly increase.

## “Random walking” from the N-terminus

The two histograms, shown in Fig. 4 for FL11-Ni and FL11-Ni-NTA, have characteristics expected for a “diffusion” distance by random walking: the nearer the zero-separation the higher the peak. These are the results of a combination of factors described below. And the high frequencies found for separations shorter than 40 Å appear to be not unreasonable, compared with the crystal structure of FL11.6),10)

In each crystal dimer of FL11, a pair of N-termini are separated by ~40 Å6) (Fig. 1c). In fact, the coordinates of four residues at the N-terminal end were not determined and thus are missing from this crystal structure. In addition, in the His-tag ten residues are inserted between the run of His residues and the N-terminal end of the protein. These missing fourteen residues will be “walking” semi-randomly under the various restrictions (e.g. the allowed ranges of the dihedral angles around peptide bonds), with a step length of ~3.3 Å, i.e. the separation between two C***α*** atoms bridged by a peptide bond. In addition, the determined part of the N-terminus, although they are rigid in the crystal, might have some flexibility in solution. In addition to the above, and most importantly, in amorphous ice the protein molecules had various orientations in 3D, and the EM images are 2D projections of these 3D orientations.

The two histograms of FL11-Ni and FL11-Ni-NTA (Fig. 4) are not exactly the same. That of FL11-Ni shows a higher degree of concentration towards the zero separation, but that of FL11-Ni-NTA has a larger degree of “diffusion”. With the presence of NTA, each block coordinating Ni is bulkier, and this might have limited the approach of the His-tags to the core of a protein dimer, resulting in the larger “diffusion”.

## The labeling efficiency

One of the complications while estimating the efficiency of this labeling method is that two closely positioned nickel clusters are not distinguished from really isolated ones. The diffusion type distributions suggest that the largest numbers of paired spots should be found with almost-zero separation, but the frequencies associated with separations less than 5 Å cannot be correctly counted. Thus, the real number of pairs should be considerably larger than 60–70%, the values described in the earlier section.

The structural unit of FL11 is a dimer,6),10) and it is unlikely that the protein is found as a monomer. Thus, each isolated spot should represent not a monomer but a dimer where only a single monomer has been labeled, unless it is an unseparated pair. If we assume that 88% of the spots are paired, and the remaining 12% are real singlets, the ratio in terms of dimers will be not 88:12 but 44:12. Then the efficiency of labeling each N-terminus, n, is estimated as ~89%, and the frequency of dimers where both monomers were unlabeled, and thus “invisible”, is estimated to be very small, ~1%. The expected frequency of dimers where both monomers were labeled is n^2^, and that of dimers where single monomers only were labeled is 2n(1 − n), while that of dimers where no monomer is labeled is (1 − n)^2^; n^2^ + 2n(1 − n) + (1 − n)^2^ = [n^2^ + n(1 − n)] + [n(1 − n) + (1 − n)^2^] = n(n + 1 − n) + (1 − n)(n + 1 − n) = n + (1 − n) = 1.

From the original concentration of FL11, 2 μM, only 2.3 spots are expected for each micrograph when taken at the magnification of 155 K, even if all of the nickel ions present in the amorphous ice of an expected width of 1,500 Å could have been focused and detected. The numbers of spots observed, 20.8 ± 9.2 for FL11-Ni and 21.2 ± 7.9 for FL11-Ni-NTA, are much higher. This discrepancy, in fact, confirms an earlier observation that while preparing a specimen, protein molecules in a droplet tend to accumulate at the surface, thus in this case towards the grid.17)

Contamination by free nickel ions unbound to the protein molecules is unlikely because of the predominant pair-wise distribution. The number of spots detected with FL11 untreated with nickel was very small, not more than 3 for each micrograph taken at the magnification of 155 K and can be zero, although the precise number depends on the cut-off level of the intensity chosen.

In the electron micrographs, no regular pattern hinting at formation of higher-order structures, e.g. a disk composed of four dimers, has been identified, although such a possibility is not totally excluded.
